# The Impact of Pre-Slaughter Fasting on the Ruminal Microbial Population of Commercial Angus Steers

**DOI:** 10.3390/microorganisms9122625

**Published:** 2021-12-19

**Authors:** Christina Breanne Welch, Jeferson M. Lourenco, Darren S. Seidel, Taylor Rae Krause, Michael J. Rothrock, T. Dean Pringle, Todd R. Callaway

**Affiliations:** 1 Department of Animal and Dairy Science, University of Georgia, Athens, GA 30602, USA; Christina.Welch@uga.edu (C.B.W.); jefao@uga.edu (J.M.L.); dsseidel18@gmail.com (D.S.S.); Taylor.Krause@uga.edu (T.R.K.); dpringle@uga.edu (T.D.P.); 2 Egg Safety and Quality Research Unit, Richard B. Russell Research Center, Agricultural Research Service, USDA, Athens, GA 30605, USA; michael.rothrock@usda.gov

**Keywords:** microbiome, rumen, steer, fasting, dysbiosis, slaughter, *Blautia*, *Methanosphaera*, *Campylobacter*, *Treponema*

## Abstract

Diet impacts the composition of the ruminal microbiota; however, prior to slaughter, cattle are fasted, which may change the ruminal microbial ecosystem structure and lead to dysbiosis. The objective of this study was to determine changes occurring in the rumen after pre-slaughter fasting, which can allow harmful pathogens an opportunity to establish in the rumen. Ruminal samples were collected before and after pre-slaughter fasting from seventeen commercial Angus steers. DNA extraction and 16S rRNA gene sequencing were performed to determine the ruminal microbiota, as well as volatile fatty acid (VFA) concentrations. Microbial richness (Chao 1 index), evenness, and Shannon diversity index all increased after fasting (*p* ≤ 0.040). During fasting, the two predominant families *Prevotellaceae* and *Ruminococcaceae* decreased (*p* ≤ 0.029), whereas the remaining minor families increased (*p* < 0.001). Fasting increased *Blautia* and *Methanosphaera* (*p* ≤ 0.003), while *Campylobacter* and *Treponema* tended to increase (*p* ≤ 0.086). Butyrate concentration tended to decrease (*p* = 0.068) after fasting. The present findings support that fasting causes ruminal nutrient depletion resulting in dysbiosis, allowing opportunistic pathogens to exploit the void in the ruminal ecological niche.

## 1. Introduction

Diet is one of the main driving forces of the microbial composition within the rumen [1,2,3], and the microbes best able to utilize dietary nutrients have a competitive advantage. The ruminal bacterial population can be broadly classified into two main niches—fibrolytic and amylolytic [4]. When a ruminant is fed a diet mainly consisting of forages, fibrolytic bacteria tend to dominate the ruminal microbial ecosystem [5,6], whereas, when ruminants are fed a high concentrate diet, amylolytic bacteria are predominant [7]. During beef cattle production, the diet fed to cattle generally changes from forage- to grain-based during the finishing phase.

Throughout the production cycle, ruminal nutrient concentrations derived from the diet drive selective pressures on the microbial population; however, cattle are often fasted for 24–36 h prior to slaughter (i.e., lairage). During fasting, the ruminal microbial population depletes the rumen of available nutrients, altering or removing the selective pressures on the rumen microbial ecosystem. Removing selective pressure can lead to a destabilization of the microbial population, or a dysbiosis, that allows opportunistic pathogens a chance to increase their populations in the rumen. Fasting cattle also reduces gastrointestinal concentrations of volatile fatty acids (VFA), which inhibit ruminal populations of *Salmonella* and *E. coli* [8]; thus, fasting allows foodborne pathogenic bacteria to increase their populations in the rumen and feces of cattle [9,10,11]. Since cattle are often asymptomatic carriers of foodborne pathogenic bacteria such as Shiga-toxin-producing *Escherichia coli* (STEC), *Salmonella*, and *Camplyobacter* [12,13,14], fasting increases the channel by which these pathogens can enter the food chain during the slaughter process.

The objective of this study was to evaluate shifts that occur in the ruminal bacterial abundance as a result of pre-slaughter fasting, and to determine if fasting resulted in ruminal dysbiosis in cattle. We hypothesized that removal of nutrient- and VFA-driven selective pressure during fasting would result in dysbiosis that could allow pathogenic bacteria the opportunity to expand their populations in the gastrointestinal tract, ultimately compromising food safety.

## 2. Materials and Methods

### 2.1. Animal Selection and Handling

All steers in this study were handled according to the guidelines approved by the University of Georgia’s Animal Care and Use Committee (AUP #A2012 11-006-R1). Steers were reared on a pasture-based system for their first year at the Northwest Georgia Research and Education Center, located in Calhoun, GA (34° 30′′ N, 84° 57′′ W). After weaning and backgrounding, a total of 63 steers were transported to a commercial feedlot located in Brasstown, NC (35° 10′′ N, 83° 23′′ W). The animals were then transitioned to a feedlot-finishing ration using a step-up approach for 3 weeks. Following transition, the steers were fed a finishing diet for 110 days. The finishing diet provided 14.51% crude protein, 2.10 Mcal/kg NEm, 1.43 Mcal/kg NEg, 0.70% Ca, and 0.45% P.

During the feedlot-finishing period, the steers were fed using a GrowSafe System (GrowSafe Systems Ltd., Calgary, AB, Canada), which measured the individual feed intake of each steer. Intake data, along with animal performance data, allowed for calculation of the residual feed intake (RFI) for each steer. An RFI value is a measure of feed efficiency calculated by subtracting the actual feed intake from the expected feed intake, where a negative RFI value means the steer is eating less than expected for their level of production and is more desirable to producers [15,16,17]. The steers most divergent in terms of RFI status (*n* = 17) were utilized during this study. Of this group, 8 were considered less-efficient (high-RFI) and 9 were assigned to the more-efficient group (low-RFI). These animals were transported to the University of Georgia Meat Science Technology Center, a federally inspected meat plant located in Athens, GA (33° 57′′ N, 83° 22′′ W). The steers were housed on site overnight with *ad libitum* access to water before humane slaughter the next morning.

### 2.2. Rumen Content Collection and Storage

Upon arrival at the slaughter facility, rumen fluid was individually collected from all steers following the procedures described in Lourenco et al. [18]. Briefly, this procedure utilized esophageal tubing and a perforated metal probe, which is attached to an electric vacuum pump for collection of the ruminal contents. Approximately 350 mL of ruminal contents was collected from each animal. Immediately after, a subsample of 45 mL was transferred to a sterile conical tube, and the tubes were stored in a −20 °C freezer until further processing. The following morning (16 h after the first collection, and after 24 h of fasting), after slaughter and evisceration of the carcasses, rumens were identified and cut into using a flame-sterilized scalpel. Approximately 45 mL of rumen contents was aseptically removed from the rumen and placed into sterile conical tubes and immediately taken to a −20 °C freezer for long-term storage until DNA extraction and volatile fatty acid (VFA) analysis were performed approximately four months later.

### 2.3. DNA Extraction and Sequencing

Bacterial DNA extraction was performed on the ruminal samples using a hybrid protocol described by Rothrock et al. [19] utilizing mechanical and enzymatic means of extraction to optimize results. Briefly, this procedure used either 330 µL of rumen fluid (pre-lairage) or 330 mg of rumen contents (post-lairage) placed in 2 mL Lysing Matrix E tubes (MP Biomedicals LLC, Irvine, CA, USA). The tubes were homogenized using a FastPrep 24 Instrument (MP Biomedical LLC, Irvine, CA, USA) in order to mechanically disrupt the cells. Enzymatic extraction occurred with the use of InhibitEX Tablets (QIAGEN, Venlo, The Netherlands). DNA from each sample was eluted and purified using an automated robotic workstation (QIAcube; QIAGEN, Venlo, The Netherlands). After extraction was completed, the concentration and purity of the DNA within each sample was measured spectrophotometrically using the Synergy H4 Hybrid Multi-Mode Microplate Reader along with the Take3 Micro-Volume Plate (BioTek Instruments Inc.; Winooski, VT, USA). In order to have sequencing performed, each sample had to meet a minimum requirement of 20 µL of volume present with a DNA concentration of at least 10 ng/µL. Samples failing to meet these minimum requirements were discarded and a new DNA extraction cycle was performed.

16S rRNA gene sequencing was performed at the Georgia Genomics and Bioinformatics Core. The library was prepared using PCR replications with the forward primer: S-D-Bact-0341-b-S-17 (5′-CCTACGGGNGGCWGCAG-3′) and the reverse primer: S-D-Bact-0785-a-A-21 (5′-GACTACHVGGGTATCTAATCC-3′) [20]. An Illumina MiSeq v3 2 × 300 base pairs kit (Illumina Inc., San Diego, CA, USA) was utilized for the 16S rRNA gene sequencing.

At the completion of sequencing, the data were demultiplexed and converted to FASTQ files. BBMerge Paired Read Merger v37.64 was used to set and merge pair-end reads. The files were then analyzed using QIIME pipeline v1.9.1 [21]. The data files were quality filtered and merged into a single file and converted to the FASTA format. Sequences were grouped into operational taxonomic units (OTU) at 97% similarity using the Greengenes database (gg_13_8_otus). Singletons were excluded from the analysis. The sequence depth was set at 17,542 sequences per sample for further analysis. In addition to the quality control steps implemented by Illumina, the quality of the sequence calls was performed using QIIME’s standard “split_libraries_fastq.py” script. Following OTU picking, sequences were aligned using the PyNAST method, which aligns sequences to a Greengenes template that is free of chimeric sequences [22]. Cyanobacterial and mitochondrial 16S rRNA genes were excluded from the analysis.

### 2.4. Volatile Fatty Acid Analysis

Analysis of VFA was performed following the procedures previously described by Lourenco et al. [23]. For the rumen fluid samples collected when the steers arrived at the slaughter facility, the samples were thawed and vortexed for 30 s to produce homogenized samples. Then, 1.5 mL of each sample was pipetted into a centrifuge tube. For the rumen contents collected at slaughter, the tubes were thawed, and 1 g of rumen contents was diluted with 3 mL of distilled water and placed into 15 mL conical tubes. The conical tubes were vortexed for 30 s to homogenize the samples, and 1.5 mL of the mixture from each tube was transferred into new centrifuge tubes. The tubes were centrifuged at 10,000× *g* for 10 min. For each sample, 1 mL of supernatant was then transferred to a new centrifuge tube and combined with 200 µL of a metaphosphoric acid solution (25% *w*/*v*). Each sample was vortexed for 30 s to ensure proper mixture and then stored at −20 °C overnight. The following morning, samples were thawed and centrifuged at 10,000× *g* for 10 min. The supernatant was then placed into polypropylene tubes with ethyl acetate in a 2:1 ratio of ethyl acetate to supernatant. The samples were vortexed for 10 s and allowed to settle for 5 min to optimize separation. Next, 600 µL of the top layer was transferred into screw-thread vials for analysis of the VFA concentrations. A Shimadzu GC-2010 Plus gas chromatograph (Shimadzu Corporation, Kyoto, Japan) with a flame ionization detector and a capillary column (Zebron ZB-FFAP; 30 m × 0.32 mm × 0.25 µm; Phenomenex Inx., Torrance, CA, USA) was used for VFA analysis. This equipment utilized helium as the carrier gas. Sample injection volume was 1.0 µL. The column temperature started at 110 °C and gradually increased to 200 °C. The injector and detector temperatures were set to 250 °C and 350 °C, respectively.

### 2.5. Statistical Analysis

Statistical analysis was performed using Minitab v.19.1. Differences in the alpha diversity indexes and bacterial relative abundances upon arrival at the slaughter facility and after slaughter were calculated using paired t-tests with the model:

$$\;t=\frac{\bar{d}-\mu_{d_{0}}}{s_{\bar{d}}}$$

where $\bar{d}$ is the sample mean difference, $\mu_{d_{0}}$ is the hypothesized population mean difference, $s_{\bar{d}}$ = $s_{\bar{d}}/\surd n$, n is the number of samples differences, and $s_{\bar{d}}$ is the standard deviation of the sample difference. Several bacterial taxa were found to be significantly different as a result of pre-slaughter fasting; however, selection of bacterial taxa presented was based on the ones found in the literature to potentially pose a threat to human or host health. In addition, multiple Pearson correlations were performed between bacterial abundances and VFA concentrations. Based on the magnitude and significance of the correlations (*p* < 0.05), linear regression analyses were performed between selected bacterial taxa and VFA concentrations using the method of least squares. Results were considered significant at *p* ≤ 0.05 and treated as trends when 0.05 < *p* ≤ 0.10.

## 3. Results

Pre-slaughter fasting dramatically impacted the overall alpha-diversities of the ruminal microbial ecosystem (Table 1). For the high-RFI (low efficiency) steers, there was an increase in bacterial richness (Chao 1; *p* = 0.001), evenness (*p* = 0.040), and diversity (Shannon index; *p* = 0.001) in the rumen samples collected at lairage compared to those at slaughter. Similarly, bacterial richness (Chao 1), evenness, and diversity (Shannon index) were higher (*p* ≤ 0.03) in the rumen of low-RFI (high efficiency) steers at slaughter compared to lairage. Since the impact of pre-slaughter fasting displayed similar patterns in bacterial populations regardless of feed efficiency status, high- and low-RFI steers were pooled (*n* = 17) for further analyses.

**Figure 1:**
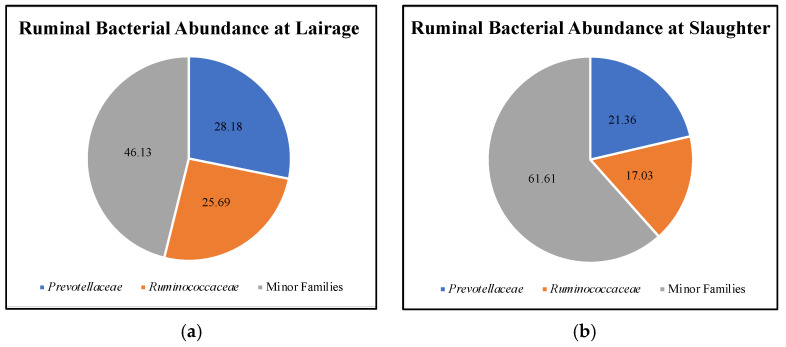
Relative bacterial abundance in the rumen of Angus steers (*n* = 17) observed at lairage (**a**) and, after fasting overnight, at slaughter (**b**) (after fasting overnight). Family abundance differed after fasting (*p* ≤ 0.03).

The taxonomic profile at the phylum and family level can be found in the supplementary material (Figure S1). All the bacterial families were combined, except for the two most abundant families, *Prevotellaceae* and *Ruminococcaceae*, which comprised greater than 53% of the total relative abundance upon arrival (Figure 1). However, after fasting, the abundance of *Prevotellaceae* decreased from 28.18 to 21.36% (*p* = 0.029) and *Ruminococcaceae* decreased from 25.69 to 17.03% (*p* = 0.002) for a combined 38.39% of the ruminal environment. Consequently, the bacterial abundance of the minor families (e.g., *Lachnospiraceae,* Order Clostridiales, S24-7, and *Veillonellaceae)* was increased from 46.13 to 61.61% (*p* < 0.001) during the overnight pre-slaughter fasting period.

**Figure 2:**
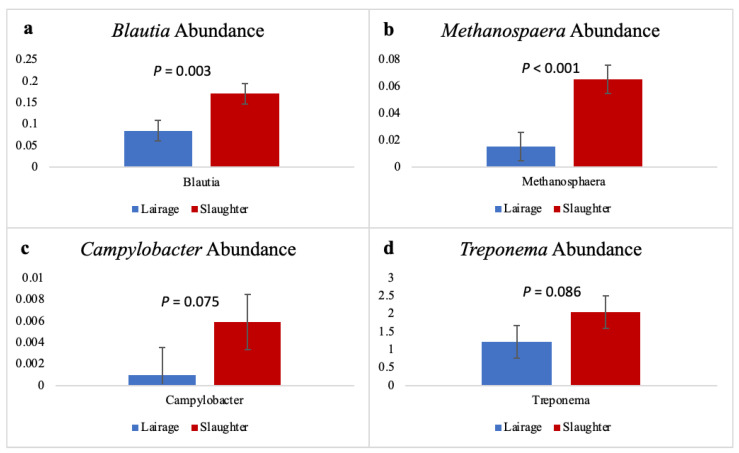
Relative bacterial abundances of: (**a**) *Blautia*, (**b**) *Methanosphaera,* (**c**) *Campylobacter*, and (**d**) *Treponema* in the rumen of Angus steers (*n* = 17) before (lairage) and after (slaughter) 16 h pre-slaughter fasting occurred. Error bars indicate the standard error of the mean. *p*-values indicate the difference between bacterial abundance from lairage to slaughter.

At the genus level, two well-known foodborne pathogens, STEC and *Salmonella*, were not detected in the rumen of steers before or after pre-slaughter fasting. However, multiple bacterial genera that contain harmful or undesirable species for ruminant animals were detected in a greater abundance after fasting. *Blautia* (*p* = 0.003; Figure 2a) and *Methanosphaera* (*p* < 0.001; Figure 2b) significantly increased in relative abundance in the rumen as a result of fasting. *Campylobacter* tended to increase (*p* = 0.075) in the rumen of steers after fasting (Figure 2c). Additionally, the genus *Treponema* tended to increase (*p* = 0.086) in abundance after pre-slaughter fasting (Figure 2d).

VFA concentrations before and after pre-slaughter fasting are demonstrated in Table 2. Fasting did not show any significant impact on ruminal VFA concentrations (*p* > 0.05). Butyrate concentrations, however, exhibited a decreasing trend in concentration after fasting from lairage to slaughter (*p* = 0.07).

**Figure 3:**
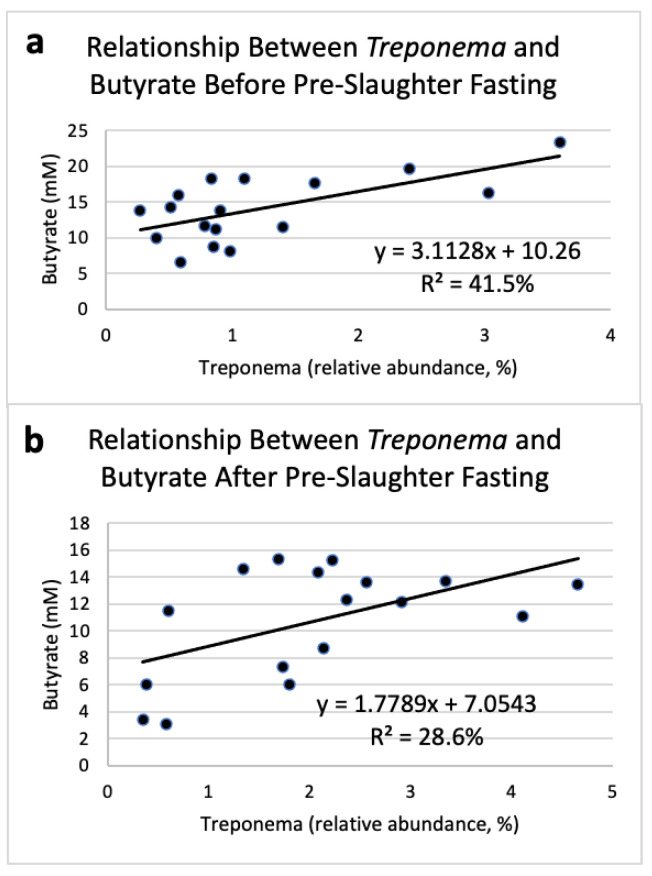
Linear regression expressing the relationship between *Treponema* relative abundance and butyrate concentration prior to pre-slaughter fasting (**a**) and after 16 h of pre-slaughter fasting (**b**) in the rumen of Angus steers (*n* = 17).

The relative abundance of *Treponema* was positively correlated with ruminal butyrate concentrations. As butyrate concentrations increased before pre-slaughter fasting, *Treponema* relative abundance also increased (R^2^ = 41.5%; Figure 3a). Similarly, butyrate concentrations in the rumen were related to the relative abundance of *Treponema* in the rumen of steers after fasting (R^2^ = 28.6%; Figure 3b).

## 4. Discussion

Ruminal microbial populations of steers were significantly influenced by pre-slaughter fasting with bacterial richness, evenness, and diversity all increasing as a result of fasting. These results highlight the immense effect fasting has upon the rumen bacterial populations. Normally, with a regular flow of nutrients into the rumen, ecological niches are filled by the bacteria best able to utilize nutrients provided by the diet [3,24,25]. In the present study, however, fasting removed dietary selective pressure in the rumen and resulted in the number of bacterial species (richness) being increased, and the distribution of these species being more equal (evenness), ultimately increasing microbial diversity in the rumen post-fasting.

*Blautia* is a carbohydrate-fermenting bacterium normally found throughout the GIT of young calves; however, its relative abundance decreases as calves age [26]. Additionally, *Blautia* populations increased in abundance in the rumen of cattle fed a grain-based diet compared to those fed a forage-based diet [27], which may indicate their ability to scavenge available soluble carbohydrates in the rumen when more numerous bacterial populations are reduced. This can potentially impact food safety since Zeineldin et al. [28] found that along with other pathogenic bacteria, this bacterial genus increased in the feces of feedlot cattle displaying hemorrhagic diarrhea. Unfortunately, the present study’s inability to detect many known foodborne pathogenic bacteria in the rumen prevents a similar correlation from being found. Still, these bacteria’s association with disease and pathogen incidence could indicate a connection between dysbiosis in the ruminal microbial population and opportunistic pathogen colonization.

Ruminal methanogens, such as *Methanosphaera*, are generally thought to waste dietary energy in the rumen that would normally be utilized by the animal and can represent a loss of up to 12% of carbon and energy in the diet [29]. *Methanosphaera* produces methane in the rumen via hydrogen-dependent reduction of methanol [30]. Furthermore, *Methanosphaera* has been found in greater abundance in inefficient steers compared to efficient steers based on RFI [31]. Additionally, it was found to be increased in humans with inflammatory bowel disease compared to healthy humans [32]. Thus, the ruminal increase in the relative abundance of *Methanosphaera* after pre-slaughter fasting may negatively affect the host by redirecting nutrients away from propionate production and ultimately contributing to host inefficiency and a decrease in host health.

*Campylobacter* and Non-typhoidal *Salmonella* are the main causes of foodborne illnesses in the United States, England and Wales, and Australia [33]. *Campylobacter* is responsible for 9% of the foodborne illnesses in the United States and accounts for 15% of total foodborne-related hospitalizations [34]. The total direct and indirect cost of each human case of *Campylobacter* is estimated to be USD 1846 [35]. Ruminal *Campylobacter* populations tended to be higher following fasting, indicating that this opportunistic foodborne pathogenic bacterium can increase in abundance during pre-slaughter fasting, which increases its chance of entering the food chain.

The relative abundance of the genus *Treponema* had a tendency to increase after pre-slaughter fasting. Although Tajima et al. [1] found ruminal concentrations of *Treponema bryantii* decreased when diet was changed from forage to grain, previous results showed that after a 16 h incubation period, there was an increase in ruminal Spirochaetes driven largely by an increase in *Treponema* [36]. These results suggest that although diet can impact the abundance of *Treponema*, this bacterium can increase in abundance if the pressure of the diet on the microbial population is removed completely. *Treponema* was positively correlated to butyrate concentrations before and after fasting, further indicating *Treponema’s* ability to scavenge available nutrients present at low concentrations. While *Treponema* has been shown to interact with cellulolytic bacteria in the rumen [37], this genus also contains many pathogenic species. Numerous studies have found multiple species of *Treponema* associated with digital dermatitis in dairy cattle and sheep [38,39,40]. Additionally, Sullivan et al. [41] found the GIT was a reservoir for pathogenic *Treponema* species in both beef cattle and sheep. Therefore, *Treponema* could be an opportunistic pathogen that benefits from removal of the selective pressure of the diet.

Acetate, propionate, isobutyrate, isovalerate, valerate, and total VFA concentrations were not changed by pre-slaughter fasting. However, butyrate concentration had a tendency to decrease after pre-slaughter fasting. These findings suggest that although there was not a regular flow of nutrients, the ruminal bacteria scavenged nutrients from an increasingly nutrient-sparse environment and continued to produce energy sources (VFA) for the host animal. Although the decrease in total VFA concentration due to fasting in our study was small, these findings are similar to other studies, which found ruminal VFA concentrations decreased with prolonged fasting [42,43]. In the present study, butyrate concentrations tended to decrease in the rumen due to fasting. Butyrate is known as the “healthy gut” VFA because GIT epithelial cells utilize it as an energy source to keep the gut functioning properly [44]. Decreased butyrate concentrations could indicate that the minor bacterial families that increased in abundance after fasting do not produce as much butyrate as those found in higher abundance prior to pre-slaughter fasting.

With the current lack of data available on the effects of pre-slaughter fasting on ruminal microorganisms, this preliminary study lays the groundwork into how the relative abundances of microbes in the rumen change as a result of pre-slaughter fasting. Although the data provide valuable insight into how ruminal dysbiosis can introduce harmful bacteria into the ruminal microbiota, this preliminary study still has some limitations. One of these limitations is due to the technology available for the small-scale study, limiting results to relative bacterial abundances. Future studies need to be conducted using qPCR in order to determine the exact quantity of each bacterial species highlighted in this study to determine their risk to human and animal health. Since the major foodborne pathogens (e.g., STEC and *Salmonella*) are typically most associated with the hindgut of cattle, further studies need to be conducted to see how pre-slaughter fasting affects other portions of the ruminant gastrointestinal microbiota to determine if the increase in harmful bacteria in the rumen would also occur in the hindgut. Although the sample size was relatively small, our findings highlight the importance of selection pressure provided by the diet and the negative impacts of the removal of the selective pressure on the host animal’s microbial ecology and, consequently, on the meat industry as a whole. Additionally, elucidating which microbial species contribute to the changes in relative microbial abundances after fasting can shed light on concerns about the possibility of fasting, contributing to an increase in harmful ruminal pathogens.

## 5. Conclusions

Collectively, our results showed that as steers were fasted prior to slaughter, their ruminal microbial consortium underwent major fluctuations. The total number of bacterial species increased, along with an increase in evenness and overall diversity of the ruminal microbiota. This shift was partially explained by the two most abundant bacterial families (*Prevotellaceae* and *Ruminococcaceae*), which decreased after fasting and created niches for other opportunistic and potentially harmful bacteria. Furthermore, the minor families prior to fasting increased in abundance after fasting. Additionally, many bacterial genera found to have deleterious effects in cattle, humans, or the environment increased after fasting, including *Blautia, Methanospeara, Campylobacter,* and *Treponema*. These results suggest that when the selection pressure applied to the ruminal microbes by the diet is removed, the microbial consortium experienced dysbiosis providing pathogens an opportunity to thrive in the rumen and, ultimately, harm the environment or enter the human food chain.

## Figures and Tables

**Table 1:** Ruminal microbial alpha-diversity of high-RFI (*n* = 8) and low-RFI (*n* = 9) steers observed at lairage and slaughter (after fasting overnight).

**Index**	**High-RFI Steers**			
	**Lairage**	**Slaughter**	**SEM**	***p*-Value**
Chao 1	2791	3786	177	0.001
Shannon Index	8.18	8.59	0.072	0.001
Evenness	0.768	0.786	0.007	0.040
	**Low-RFI Steers**			
	**Lairage**	**Slaughter**	**SEM**	***p*-Value**
Chao 1	2499	3051	204	0.027
Shannon Index	7.92	8.45	0.161	0.011
Evenness	0.753	0.788	0.014	0.030

**Table 2:** Ruminal volatile fatty acid concentration (m*M*) of commercial Angus steers (*n* = 17) at lairage and slaughter (after fasting overnight for 16 h).

Volatile Fatty Acid	Lairage	Slaughter	SEM	*p*-Value
Acetate	62.50	62.73	6.740	0.97
Propionate	26.13	22.50	4.520	0.43
Isobutyrate	1.29	1.27	0.081	0.88
Butyrate	14.06	10.71	1.710	0.07
Isovalerate	3.27	3.70	0.397	0.30
Valerate	1.71	1.76	0.467	0.91
Total volatile fatty acid	109.21	103.56	13.100	0.67
Acetate: Propionate	2.63	3.18	0.332	0.12

## Data Availability

The data have been made publicly available, and readers can find it at: https://www.mg-rast.org (accessed on 1 November 2021) using the accession number: mgm4909317.3.

## Supplementary Materials

The following are available online at https://www.mdpi.com/article/10.3390/microorganisms9122625/s1, Figure S1: Taxonomic profiles at the phylum (a) and family (b) levels of the relative bacterial abundance of the rumen at lairage and slaughter of Angus steers (*n*=15).
