# Supplementary material for: The Impact of Pre-Slaughter Fasting on the Ruminal Microbial Population of Commercial Angus Steers
Source: Microorganisms. 2021 Dec 19;9(12):2625. doi: 10.3390/microorganisms9122625 (PMC8709334; doi:10.3390/microorganisms9122625)
Supplement: Supplementary file 1 [file microorganisms-09-02625-s001.zip › microorganisms-1503976-supplementary.pdf]

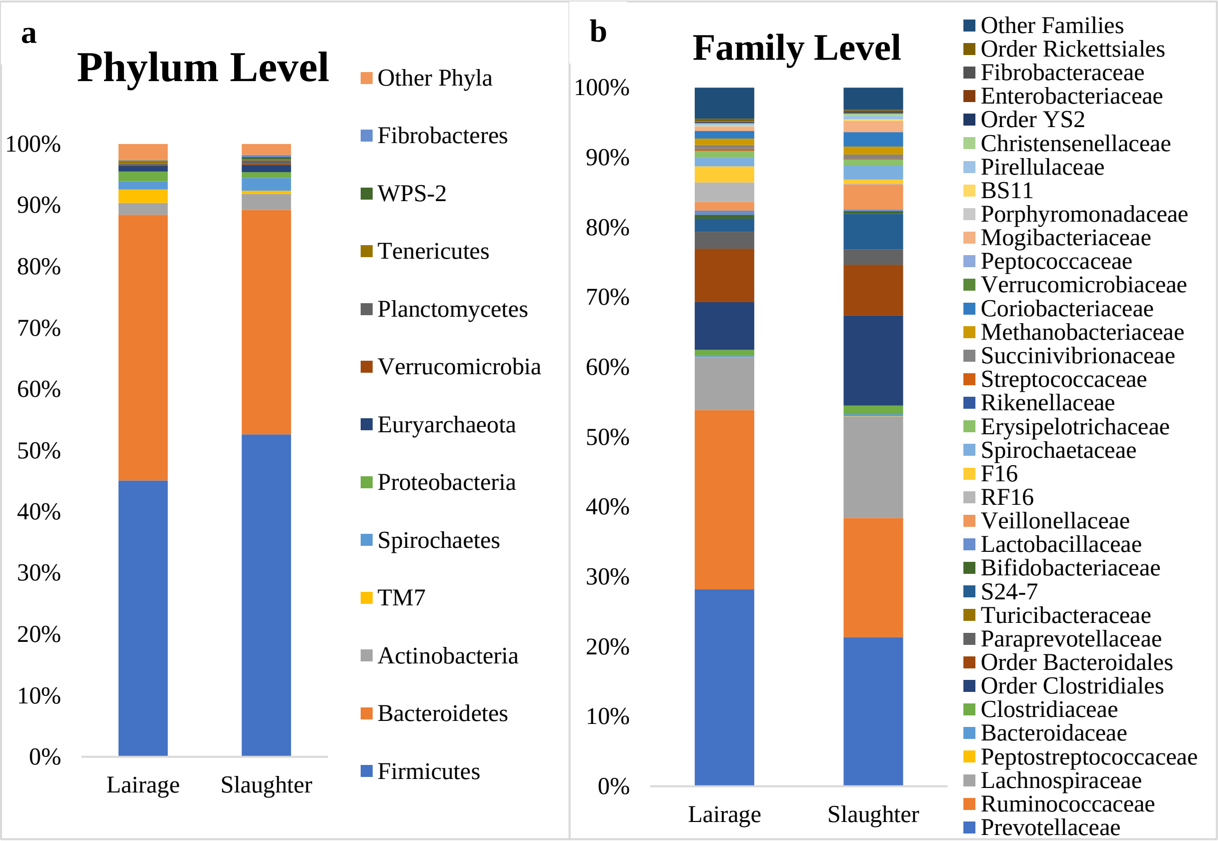

Supplemental Figure S1. Taxonomic profiles at the phylum (**a**) and family (**b**) levels of the relative bacterial abundance of the rumen at lairage and slaughter of Angus steers (n=15).
